# Global mental health: trauma and adversity among populations in transition

**DOI:** 10.3402/ejpt.v7.31140

**Published:** 2016-02-12

**Authors:** Brian J. Hall, Miranda Olff

**Affiliations:** 1 Global and Community Mental Health Research Group, Department of Psychology, Faculty of Social Sciences, University of Macau, Macau (SAR), People’s Republic of China; 2 Department of Psychiatry, Academic Medical Center, University of Amsterdam, Amsterdam, The Netherlands, Arq Psychotrauma Expert Group, Diemen, The Netherlands

Migration and trauma is a critical and timely area of inquiry given the enormous transnational migration occurring within the past year. The UN estimates there are over 244 million people living outside of their country of origin (United Nations Population Fund, 2016). United Nations High Commissioner for Refugees (UNHCR) estimated there were over 1,000,000 refugees fleeing to Europe by boat during the year 2015 (UNHCR, 2016). The nature of their migration is forced and due to war and violence in their home countries (e.g., Turner, 2015). Studies indicate that refugee communities are at increased risk for common mental disorders including posttraumatic stress disorder (PTSD), depression, and anxiety (Lindert, Von Ehrenstein, Priebe, Mielck, & Brähler, 2009). Therefore, the mental health of this sizable population in Europe and in other countries in the Middle East and globally is an important public mental health priority. Traumatic event exposure may occur during three key phases: pre-migration, transit, and post-migration (Zimmerman, Kiss, & Hossain, 2011). Continued research into the consequences of trauma exposure along this continuum, and intervention and prevention programs addressing the mental health of individuals and communities in transition are needed.

This special issue of the *European Journal of Psychotraumatology* offers a collection of papers that focus on migrating populations experiencing trauma and adversity. These papers focus on several key issues facing migrant populations including anthropometric indicators of children exposed to violence in South Africa (Koen et al., 2016); the effects of acculturative stressors on refugees in Australia and Austria (Kartal & Kiropoulos, 2016); social integration and psychological impairment in traumatized refugees (Schick et al., 2016); prevalence of PTSD and depression among Iraqi Yazidi refugees (Tekin et al., 2016); organized violence as predictors of aggression and violent behaviors among refugees (Mueller-Bamouh, Ruf-Leuschner, Dohrmann, Schauer, & Elbert, 2016); psychological treatment for refugees (Ter Heide, Mooren, & Kleber, 2016); and posttraumatic growth among refugee adolescents (Sleijpen, Haagen, Mooren, & Kleber, 2016).

The paper by Koen et al. (2016) examined the associations between maternal trauma and posttraumatic stress disorder and adverse birth outcomes among 544 mother–infant dyads in the Drakenstein Child Health Study in South Africa. The results of their study indicated that an estimated prevalence of current and lifetime posttraumatic stress disorder was 19% and that showed that recent life stressors were associated with PTSD. Mothers’ traumatic event exposure was significantly associated with reduced infant head circumference, indicating that trauma affected fetal growth and development. This study importantly illustrates the potential for intergenerational effects of trauma on a population in transition.

Tekin and colleagues contribute a novel investigation into gender-based differences in the prevalence of PTSD and depression among Iraqi Yazidi refugees (Tekin et al., 2016). A total sample of 238 were assessed using a structured clinical interview. The prevalence of PTSD was 42.9%, 39.5% for major depression, and 26.4% for comorbid PTSD and major depression. Similar to previous research, women reported higher prevalence of PTSD and depression. The authors suggest that over- or under-modulation of emotions plays a critical role in explaining these findings among refugees.

Research has demonstrated that childhood family traumas may influence later delinquency. Mueller-Bamouh and colleagues (2016) explore the role of family trauma and appetitive aggression as independent predictors of aggressive acts among 49 male unaccompanied minor refugees. The results indicated that in addition to exposure to family violence, trait-level aggression was a stronger predictor of aggressive behavior. Exposure to organized, state-sponsored violence was associated with increased PTSD, but not aggression. This study suggests that in addition to family difficulties, children may also have an underlying vulnerability to aggression that should be included in treatment planning. Future longitudinal studies are needed to explore these associations among refugee children.

There is an established link between trauma exposure and common mental disorders among refugee populations settling in high-income contexts. Post-migration stressors are also key determinants for poor mental health (Stotz, Elbert, Müller, & Schauer, 2015). Two papers in our series explore these factors. Kartal and Kiropoulos (2016) examine the role of acculturative stress in 138 Bosnian refugees living in Austria and Australia. After controlling for potential confounders, acculturative stress was associated with increased PTSD and anxiety symptoms among refugees living in Austria. In Australia, traumatic event exposure, but not acculturative stress, was associated with increased PTSD, anxiety, and depressive symptoms. This study highlights the importance of the receiving country context in shaping mental health outcomes for refugees.

Social integration is also a key post-migration factor associated with poor mental health outcomes. Schick and colleagues explore the role of social integration and psychological impairment among 104 treatment-seeking refugees in two clinics in Switzerland (Schick et al., 2016). Their findings suggest that symptoms of psychological distress were more strongly associated with post-migration living difficulties (e.g., communication difficulties) than individual-level factors such as education and structural determinants like their visa status.

The timing of the appropriate application of evidence-based treatments for refugees is a critical concern (Acarturk et al., 2015). Ter Heide et al. (2016) explore the nature of complex PTSD among refugees and offer a cogent analysis of its role of trauma treatment. They advance the position that only few refugees develop complex PTSD and that trauma-focused treatment should be delivered to all refugees who are seeking treatment for PTSD. Their debate piece stimulates us to consider whether phased treatments do more harm than good, and remind us that trauma-focused treatment, applied consistently and following prescribed guidelines, can greatly benefit refugees (see also Cloitre, 2015).

Finally, Sleijpen and colleagues (2016) offer a unique look at the association between posttraumatic growth and dispositional optimism, perceived social support, and PTSD among 111 refugee and asylum-seeking adolescents. At least some PTG was reported by the sample. PTG and PTSD were not statistically significantly associated, but PTG was associated with higher levels of optimism and social support. This is one of the first studies conducted to explore these associations within an adolescent population and future research, especially longitudinal (e.g., Hall, Saltzman, Canetti, & Hobfoll, 2015) is needed to tease apart the role of PTG in their lives.

This special issue focuses our attention on key issues faced by forced migrants in different global regions, which builds on the special issue on Global Mental Health and Trauma published in 2015 (Purgato & Olff, 2015). With continued political and economic instability, the substantial increase in world migration in 2015 will likely continue to rise. Research into the key determinants of migrant mental health and potential opportunities to improve their condition has become a pressing concern. We hope this series of articles will stimulate continued research and policy-level discussions to improve the mental health of this vulnerable population.

Brian J. Hall Global and Community Mental Health Research Group Department of Psychology Faculty of Social Sciences University of Macau Macau (SAR), People's Republic of China Department of Health, Behavior, and Society Johns Hopkins Bloomberg School of Public Health Baltimore, MD, USA E-mail: brianhall@umac.mo; bhall31@jhu.edu Miranda Olff Department of Psychiatry Academic Medical Center University of Amsterdam Amsterdam, The Netherlands Arq Psychotrauma Expert Group Diemen, The Netherlands
